# Total knee arthroplasty using trochlear groove as guide for position of femoral component in severe knee osteoarthritis

**DOI:** 10.1186/s12893-016-0148-z

**Published:** 2016-05-23

**Authors:** Gangyong Huang, Jun Xia, Siqun Wang, Yibing Wei, Jianguo Wu, Feiyan Chen, Jie Chen, Jingsheng Shi

**Affiliations:** Department of Orthopaedic Surgery, Huashan Hospital Fudan University, 12th Wulumuqi Middle Road, Shanghai, 200040 China

**Keywords:** Total knee arthroplasty, Knee osteoarthritis, Patellar tracking, Trochlear groove, Prosthesis

## Abstract

**Background:**

Apart from transepicondylar axis, the native femoral sulcus was also reported to be used as a guide for the femoral component position in total knee arthroplasty (TKA). However, it was not shown in patients with severe knee osteoarthritis. This study was conducted to compare the position of trochlear groove in patients with and without osteoarthritis, and to assess whether trochlear groove could be used as a guide for position of femoral component in TKA for severe knee osteoarthritis.

**Methods:**

Total 50 severe knee osteoarthritis patients (Kellgren Lawrence grade 3 or 4) who underwent TKA were included. Meanwhile, 50 patients who underwent arthroscopic surgery without osteoarthritis were included as control. The distance from trochlear groove to the midpoint of a virtual anterior condyle osteotomy line (parallel to the posterior condyle line) (a–b) was recorded by radiological and surgical measurements. Midpoint of transepicondylar axis and trochlear groove were used as guide for placing prosthesis model in TKA, respectively. No-thumb test was performed to assess the patellar tracking. The position of femoral component was finally performed using trochlear groove as guide in TKA.

**Results:**

Value of “a–b” was significantly different between osteoarthritic and control knees (*P* = 0.008). During the placement of prosthesis model, similar patellar tracking was detected between using midpoint of transepicondylar axis and trochlear groove as guide (*P* > 0.05). After placing femoral component using trochlear groove as guide, most patients obtained good patellofemoral congruence with pneumatic tourniquet inflated (*n* = 43) or deflated (*n* = 5), and good patellofemoral congruence was also obtained by lateral patellar retinaculum release in two patients.

**Conclusion:**

Despite the shifting of trochlear groove caused by severe knee osteoarthritis, trochlear groove can be used as a guide for position of femoral component, with equivalent patellar tracking compared with transepicondylar axis.

## Background

Total knee arthroplasty (TKA) is a well-proven procedure with success rate of more than 90 % [1, 2]. However, patellofemoral complications (such as anterior knee pain, patellar subluxation, patellofemoral wear and patellar prosthesis loosening and fracture) commonly occurred after TKA, which could lead to poor function of the knee [3]. Previous studies have found that the operative technique, prosthetic design, axial malalignment and malrotation as well as poor patellar tracking contributed to the patellofemoral complications [4–7]. Besides, femoral component designs and position were also reported as the main causes of poor patellar tracking in TKA [8–10].

Currently, transepicondylar axis (also known as the surgical epicondylar axis), a line between the medial and lateral epicondyles (3° of external rotation relative to the femur posterior condyles line), is commonly used as a guide for positioning of the femoral component in TKA [11, 12]. Compared with other reference lines (such as Whiteside's line, femoral transverse axis or posterior condylar axis), the transepicondylar axis was recommended for valgus knees [13–15]. However, it was reported that only 75 % of knees would have been within 3° of the true epicondylar axis when using epicondyles to control rotation, so the clinical estimation of the epicondylar axis was inaccurate [16]. Moreover, transepicondylar axis was just logical and appropriate when used as a principal axis for knees between 0° and 60° of flexion [17].

Recently, previous studies have reported that native femoral sulcus could be used as a guide for the femoral component position in TKA [8, 18]. However, it was not known yet whether trochlear groove was shifting with line of force in severe osteoarthritic knee, and whether the positioning of the femoral component using trochlear groove as reference contributed to the reconstruction of patellar tracking in patients with severe knee osteoarthritis. Thus, this study was conducted to compare the position of trochlear groove in patients with and without osteoarthritis. Meanwhile, we also compared the patellar tracking by using the midpoint of transepicondylar axis and trochlear groove as guide for the position of femoral component. Our study would give the surgeons instruction of the position of femoral component during the TKA for patients with severe knee osteoarthritis.

## Methods

### Patients

Total 197 consecutive patients who underwent selective TKA for knee osteoarthritis in the Department of Orthopaedic Surgery, Shanghai Huashan Hospital from January 2009 to January 2011 were recruited in knee osteoarthritis group for the study. The inclusion criteria were as follows: age of more than 20 years old; a diagnosis of severe knee osteoarthritis (Kellgren Lawrence grade 3 or 4) according to the Kellgren and Lawrence classification criteria [19, 20]; unilateral TKA would be performed. Patient with unwillingness to sign the informed consent form or who had extra-articular malformation was excluded. All osteoarthritis patients who were scheduled for TKA took the full-length weight-bearing radiographs of both lower extremities and computed tomography (CT) scanning of the knee before surgery. Meanwhile, patients who underwent arthroscopic surgery homochronously in our department were recruited as controls. The patients in control group should meet the following criteria: age of above 20 years old; no history of knee arthropathy; no diagnosis of osteoarthritis through preoperative routine X-radioscopy and arthroscopy; and consent to receive the CT scanning to rule out osteoarthritis. This prospective study was approved by the ethics committee of Shanghai Huashan Hospital.

### The CT scanning

**Fig. 1:**
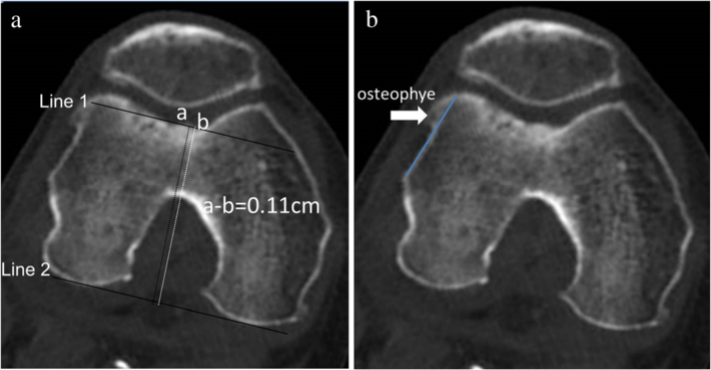
Preoperative computed tomography (CT) scanning. **a** the measurement of distances from the lowest point of trochlear groove to the midpoint of anterior condyle in CT images. Line 1: the virtual anterior condyle osteotomy line; line 2: the posterior condyle line. **b** the osteophytes in the patients with severe knee osteoarthritis

The CT scans were performed using a 256-slice multi-detector CT (MDCT) scanner (Philips Brilliance iCT, Philips Healthcare, Cleveland, USA). Patients were placed in supine position with the knee fully extending and feet closed together. All images were reconstructed with 3-mm slice thickness at 3-mm intervals in the transverse (axial) planes. Image data were then transferred to a Centricity picture archiving and communication system (PACS) workstation (Centricity 1.0; GE Healthcare) and analyzed using Centricity PACS software (GE Healthcare, Chalfont St Giles, Buckinghamshire, UK). The first layer image near intercondylar fossa was used and evaluated by two experienced orthopedic radiologists. As shown in Fig. 1a, the lowest point of femoral trochlea (trochlear groove) was defined as point “a”, and the midpoint of a virtual anterior condyle osteotomy line (which was parallel to the line of posterior condyle) was defined as point “b”; the distance from “a” to “b” (a–b) was measured. The positive value of distance a–b was determined with the “a” point at the lateral of “b” point; otherwise, the negative value of distance a–b was obtained. The measurements for 20 repeated radiographs were performed to determine intra-observer reliability, which were evaluated using an intraclass correlation coefficient (ICC, one way random).

### Operative procedure

**Fig. 2:**
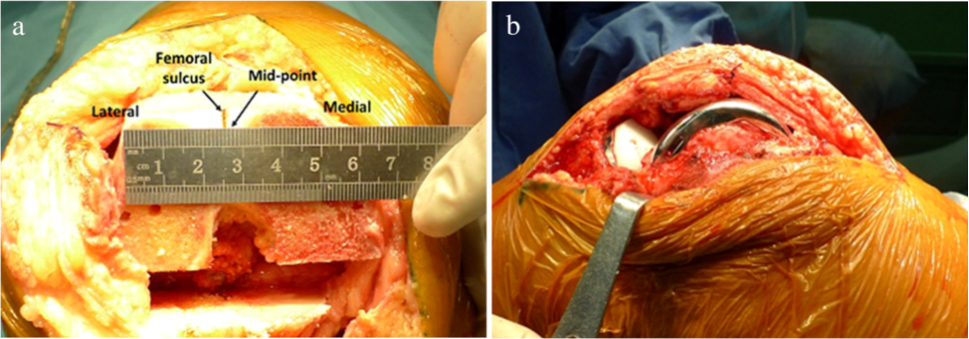
Operative procedure. **a** the measurement of distances from the lowest point of trochlear groove to the midpoint of anterior condyle osteotomy line during the surgery. **b** patellar tracking was evaluated by no-thumb test

All the osteoarthritis patients received knee surface replacement with the preservation of the patella. A senior specialist performed the TKA using Genesis II prosthesis (Genesis II PS High-Flexion, Smith and Nephew, USA) which had an intrinsic 3° of internal rotation (the medial posterior condyle was thinner than the lateral posterior condyle in this prosthesis). Patients were placed in supine position and a pneumatic tourniquet was inflated on the proximal thigh for reducing intraoperative blood loss. Under general anesthesia, the knee joint was exposed through medial parapatellar arthrotomy. After removing the osteophytes (Fig. 1b) on the femur, the distal femoral osteotomy was performed perpendicular to the mechanical axis of the femur. Then osteotomy for anterior and posterior condyle was performed based on the posterior condyle line. Subsequently, the distance from “a” to “b” was measured as shown in Fig. 2a. After oblique osteotomy and the preparation of soft tissues, a prosthesis model was placed in each patient using midpoint of transepicondylar axis and trochlear groove as reference point respectively. Meanwhile, patellar tracking was evaluated using no-thumb test [21, 22]. No-thumb test was considered positive if patellar maltracking was identified under tourniquet deflation, otherwise, negative result was considered. Finally, the femoral prostheses were fixed with the trochlear groove as reference point, and patellar tracking was re-evaluated using no-thumb test (Fig. 2b). A lateral retinacular release was performed if no-thumb test was positive.

### Postoperative management and follow-up

Patient were on bed rest with the affected limb elevated at postoperative 24 h and with ice applied to the wound site to decrease swelling and pain. Intravenous injection of Cefuroxime (1.5 g) was conducted once during postoperative 12–24 h for infection prevention. Elastic stocking was used after the removal of negative pressure drainage in case of thrombosis, and the prophylaxis of deep vein thrombosis prophylaxis was also performed by the administration of blood coagulation factor X-antagonist (Rivaroxaban) for 4 weeks. Physiotherapy was performed under the guidance of physiatrician. Patients were encouraged to take quadriceps femoris muscular training and range of motion (ROM) exercises of the knee from postoperative 1 day, and were allowed to take weight-bearing walking with additional protection. Thereafter they were allowed to take independent ambulation and daily living training at postoperative 6 week.

All the patients receiving TKA were followed-up for at least 1 year. During the follow-up, the knee society score (KSS), visual-analogue scale (VAS) score for anterior knee pain and postoperative complications were assessed and recorded.

### Statistical analysis

The sample size was estimated by using the following formula: $$ \begin{array}{l}\mathrm{N}={\mathrm{Z}}^2\times \left(\mathrm{P} \times \left(1 - \mathrm{P}\right)\right)/{\mathrm{E}}^2\\ {}\end{array} $$ [23], where *N* = sample size; *Z* = the standard normal deviation, usually 1.96 at 95 % confidence level; *P* = expected prevalence rate of 50 %; and *E* = precision (margin of error at 15 %). Based on this formula, the minimum sample size needed was 43, and power of this study was 0.9.

Data were analyzed using SPSS 15.0. The independent sample *t*-test was applied for the comparison between knee osteoarthritis and control groups. The paired *t*-test was used for the comparison of parameters before and after surgery. Comparison of proportions was performed using the *χ*^2^ test. The correlation between offset of mechanical axis and position of trochlear groove was assessed using the Pearson's correlation test. For all the analysis, *P* < 0.05 was considered statistical significant.

## Results

In this study, only 52 patients with knee osteoarthritis signed the informed consent forms, among whom one patient with rheumatoid arthritis was excluded and the other case accompanied with ipsolateral Crowe-IV developmental dysplasia of the hip were also excluded due to the severe articular valgus. Total 50 patients who underwent arthroscopic surgery homochronously were recruited as controls, including 28 cases of gonarthromeningitis, 17 cases of meniscus injury, 3 cases of knee ligament injury and 2 cases of knee joint pain caused by saphenous nerve entrapment.

### Comparison between knee osteoarthritis and control groups

**Fig. 3:**
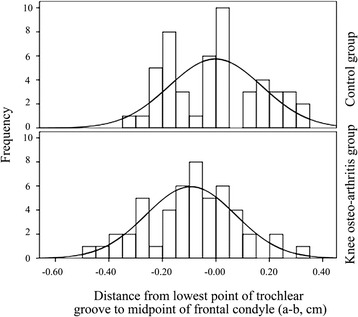
Distances from the lowest point of trochlear groove to the midpoint of virtual anterior condyle osteotomy line (a-b) by CT scanning

As shown in Table 1, knee osteoarthritis and control patients were similar in sex and affected side (*P* > 005). However, patients in knee osteoarthritis group were significantly older than control ones (*P* < 0.001). According to the CT scanning, knee osteoarthritis patients did not differ from control patients in femur anterior condyle width (*P* = 0.994), whereas there was significant difference in the value of “a–b” between the two groups (*P* = 0.008). In addition, Fig. 3 showed that the proportion of knee osteoarthritis patients with the value of “a–b” between -0.10 cm and 0.00 cm was the highest, whereas the most control patients had a value of “a–b” between 0.00 cm and 0.05 cm. Meanwhile, the results of ICC showed a significant consistency among the repeated measurements (*r* > 0.9, *P* < 0.05).

**Table 1:** Comparison between knee osteoarthritis and control group

Indexes	Knee osteoarthritis group	Control group	*P*-value
Sex (male/female)	16/34	24/26	0.102
Mean age (range), year	71.0 ± 5.8 (62–84)	45.5 ± 12.4 (22–65)	<0.001
Affected side (left/right)	33/17	29/21	0.410
Results of CT			
Width of femur anterior condyle, cm	5.41 ± 0.33	5.37 ± 0.40	0.994
	95 % CI: 5.32–5.52	95 % CI: 5.27–5.48	
Distance from “a” to “b” (a–b), cm	-0.09 ± 0.17	0.00 ± 0.17	0.008
	95 % CI: -0.04–-0.14	95 % CI: -0.47–0.52	

Data of continuous variables were shown as mean ± SD; Data of categorical variables were presented as proportions. CT: computed tomography. Distance a–b was the distance from the lowest point of trochlear groove to the midpoint of virtual anterior condyle osteotomy line

### Clinical outcomes

**Fig. 4:**
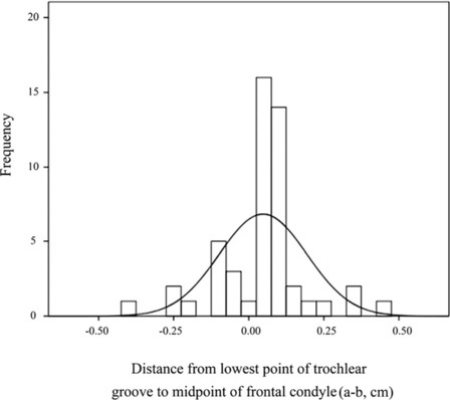
Measured distances from the lowest point of trochlear groove to the midpoint of anterior condyle osteotomy line (a–b) during the surgery

During the operation, the width of femur anterior condyle and the value of “a–b” were re-measured, and similar measurements were obtained compared with CT scanning (*P* > 0.05, Table 2 and Fig. 4). In addition, joint deformity was assessed by the full-length weight-bearing radioscopy in these patients with severe knee osteoarthritis. There were 88 % patients (*n* = 44) with varus deformity (10.2 ± 3.7°) and 12 % patients (*n* = 6) with valgus deformity (6.1 ± 5.1°). No correlation was found between the intraoperative position of trochlear groove (value of “a–b”) and the joint deformity (*r* = 0.068, *P* = 0.639).

**Table 2:** Measurements of femur anterior condyle width and distance from “a” to “b” by CT scanning and intraoperative measurement

Indexes	CT measurement	Intraoperative measurement	*P*-value
Width of femur anterior condyle, cm	5.41 ± 0.33	5.43 ± 0.43	0.200
	95 % CI: 5.32–5.52	95 % CI: 5.31–5.55	
Distance from “a” to “b” (a–b), cm	-0.09 ± 0.17	0.05 ± 0.15	0.078
	95 % CI: -0.04–-0.14	95 % CI: 0.01–0.09	

Distance a–b was the distance from the lowest point of trochlear groove to the midpoint of anterior condyle osteotomy line

The no-thumb test showed the similar results of patellar tracking by using midpoint of transepicondylar axis and trochlear groove as reference point for placing prosthesis model (*P* > 0.05, Table 3). After prostheses placement with trochlear groove as a reference point, total 43 patients had good patellofemoral congruence with the fixation of pneumatic tourniquet. Meanwhile, good patellofemoral congruence was obtained after the deflation of tourniquet in 5 patients. For the other two patients, good patellofemoral congruence was also achieved by lateral retinacular release.

**Table 3:** Results of no-thumb test

Guide for position of femoral prosthesis	Results of no-thumb test		*P*-value
	positive	negative	
Midpoint of transepicondylar axis	5	45	0.436
Trochlear groove	2	48	

During the median follow up of 2.6 years (range 1–4.25 years), the median KSS score was 92 (interquartile range: 80–104). Total 90 % (45/50) patients had VAS score lower than 3. Among other 5 patients, one patient had VAS score of 4 and four patients had VAS score of 3. One patient developed deep vein thrombosis of lower limb at 7 days and 3 weeks after surgery and was cured by conventional treatment. No other complications, such as pulmonary embolism, postoperative periprosthetic fracture, wound infection and flexion instability from malrotation, occurred in these patients during follow up.

## Discussions

It has been reported that trochlear groove was near to the midline of the medial and lateral condyles in normal knees and it would be moved during the flexion of joint [24, 25]. In the present study, trochlear groove was shown at the midpoint of a virtual anterior condyle osteotomy line (which was parallel to the posterior condyle line) of knee without osteoarthritis but was 0.9 mm medial to that midpoint in osteoarthritic knee. However, Lingaraj and Bartlett (2009) reported that the mean sulcus position was 0.7 mm lateral to the midline of the distal femoral resection in patients undergoing TKA [26]. Besides, a previous study by Eckhoff et al. (1996) demonstrated that the sulcus of the trochlear groove was also lateral to the midline but with no significant difference between osteoarthritic and normal knees [25]. The inconsistent results might be explained by the different reference line used as the midline of distal femur for the position of trochlear groove, as Lingaraj and Bartlett used the midline perpendicular to the distal margin of oblique osteotomy [26], Eckhoff et al. used the midline perpendicular to the posterior condyle line [25], and our study adopted the midline perpendicular to the distal margin of anterior condyle osteotomy. Besides, the severity of knee osteoarthritis causing joint deformity might be the other reason for the different results among the aforementioned studies. Anyhow, it indicated that the trochlear groove was significantly shifted during the occurrence of knee osteoarthritis. On the other hand, there was no significant difference in the distance a–b by using CT scanning and intraoperative measurement, suggesting that the position of trochlear groove using the distal margin of anterior condyle osteotomy as reference was stable. However, considering CT scan could not support full visualization of cartilage [27] and might produce errors between radiological and clinical determination of trochlear groove, further study was needed to verify the shift of trochlear groove in osteoarthritis and explore the mechanism.

Patients with severe knee osteoarthritis were always accompanied with varus or valgus deformity [28]. In this study, most of patients (88 %) had varus deformity and 12 % patients had valgus deformity; nevertheless, no significant correlation was found between the position of trochlear groove and the joint deformity. This was in consistent with Lingaraj and Bartlett (2009) who also found the difference in the mean sulcus position was not significant between valgus and varus knees [26].

During the surgery, the no-thumb test showed the similar patellar maltracking by using midpoint of transepicondylar axis and trochlear groove as guide for placing prosthesis model. Previous studies have showed that the position of femoral prosthesis could influence the patellar tracking [10, 18]. Also for the patellar prosthesis, it was reported that about 2.5 mm inward shifting of patellar prosthesis could improve patellar tracking [29]. In this study, TKA was performed with the preservation of the patella. Thus, the patellar tracking was mainly affected by the position and design of femoral prosthesis as well as soft tissue technique. Furthermore, it was reported that the patellar tracking was more associated with the changed position of trochlear groove in the TKA without patellar replacement [30–32]. This further supported that trochlear groove might be the most appropriate guide for position of femoral prosthesis in this study. In addition, it has been reported the slightly external rotation of femoral prosthesis was useful for improving patellar tracking in TKA [33–36]. However, when a conventional femoral component was externally rotated, some rotational incongruity may occur between the femoral and tibial articular surfaces regardless of the position of the tibial component [9]. Thus the use of a femoral component with a thicker posterolateral than posteromedial femoral condyle would minimize the rotational malalignment [37]. Despite the neutral rotation of the femoral component, good patellar tracking was also achieved after TKA by using trochlear groove as a reference.

In addition, previous study has reported that trochlear groove was not a straight line [38]. In this study, only the lowest point of trochlear groove at the distal margin of anterior condyle osteotomy (which was available for positioning) was used in this study due to the limitation of operation. Thus, more studies were required to verify the results of this study.

There were some other limitations in this study. Firstly, patients aged over 70 years old, who underwent arthroscopy but not had knee osteoarthritis, were rare, so there was a big difference in mean age of knee osteoarthritis and control groups in this study, which would affect the results. Secondly, patellar tracking after the placement of prosthesis model was only assessed using no-thumb test, and the patellar maltracking might be overestimated [39]. Third, prostheses were finally fixed with the trochlear groove as reference, and comparison of patellar tracking after the placement of prosthesis by using transepicondylar line and trochlear groove as references was not performed. Besides, most of patients (88 %) in this study had varus deformity, which may bring bias for the results of this study. Hence, further prospective randomized control study should be performed to compare the clinical outcomes of TKA using transepicondylar axis and trochlear groove as reference for knee osteoarthritis patients, as well as to confirm the accuracy of the femoral component placement using trochlear groove.

## Conclusions

In conclusion, it was found that trochlear groove was shifted during the occurrence of severe osteoarthritis. Similar patellar tracking was detected by no-thumb test when using trochlear groove and transepicondylar axis as guide for position of femoral component. Meanwhile, no correlation was found between position of trochlear groove and joint deformity. Our study would give the surgeons a reference that trochlear groove could be used as a guide for position of femoral component during the TKA for patients with severe knee osteoarthritis.

## Abbreviations

TKA, total knee arthroplasty; CT, computed tomography; MDCT, multi-detector CT; PACS, picture archiving and communication system; ICC, intraclass correlation coefficient; ROM, range of motion; KSS, knee society score; VAS, visual-analogue scale.
